# Spatio-temporal analysis of the relationship between WNV dissemination and environmental variables in Indianapolis, USA

**DOI:** 10.1186/1476-072X-7-66

**Published:** 2008-12-18

**Authors:** Hua Liu, Qihao Weng, David Gaines

**Affiliations:** 1 Department of Political Science and Geography, Old Dominion University, Norfolk, Virginia 23529, USA; 2 Center for Urban and Environmental Change, Department of Geography, Indiana State University, Terre Haute, Indiana 34709, USA; 3 Office of Epidemiology, Virginia Department of Health, Richmond, Virginia 23219, USA

## Abstract

**Background:**

This study developed a multi-temporal analysis on the relationship between West Nile Virus (WNV) dissemination and environmental variables by using an integrated approach of remote sensing, GIS, and statistical techniques. WNV mosquito cases in seven months (April-October) of the six years (2002–2007) were collected in Indianapolis, USA. Epidemic curves were plotted to identify the temporal outbreaks of WNV. Spatial-temporal analysis and k-mean cluster analysis were further applied to determine the high-risk areas. Finally, the relationship between environmental variables and WNV outbreaks were examined by using Discriminant Analysis.

**Results:**

The results show that the WNV epidemic curve reached its peak in August for all years in the study area except in 2007, where the peak was reached in July. WNV dissemination started from the central longitudinal corridor of the city and spread out to the east and west. Different years and seasons had different high-risk areas, but the southwest and southeast corners show the highest risk for WNV infection due to their high percentages of agriculture and water sources.

**Conclusion:**

Major environmental factors contributing to the outbreak of WNV in Indianapolis were the percentages of agriculture and water, total length of streams, and total size of wetlands. This study provides important information for urban public health prevention and management. It also contributes to the optimization of mosquito control and arrangement of future sampling efforts.

## Background

West Nile Virus (WNV) is a mosquito-borne disease. It was first discovered in the West Nile District of Uganda in 1937. According to the reports from the Centers for Disease Control and Prevention, WNV has been found in Africa, the Middle East, Europe, Oceania, west and central Asia, and North America. Its first emergence in North America began in the New York City metropolitan area in 1999. It is a seasonal epidemic in North America that normally erupts in the summer and continues into the fall, presenting a threat to environmental health. Its natural cycle is bird-mosquito-bird and mammal. Mosquitoes, in particular the species Culex pipiens, become infected when they feed on infected birds. Infected mosquitoes then spread WNV to other birds and mammals including humans when they bite. In humans and horses, fatal Encephalitis is the most serious manifestation of WNV infection. WNV can also cause mortality in some infected birds.

The spread of WNV has shown unique distribution patterns in different regions [1-5]. Environmental determinants, such as the presence of suitable habitats, temperatures, and climates, play important roles in WNV dissemination in North America [6,7]. Mosquito Culex species appear to prefer some land use and land cover (LULC) types (e.g., wetlands and specific grasslands) than some others (e.g., exposed dry soils). Mosquitoes in the canopy site are believed to possess more infections than those in subterranean areas and on the ground [8]. Wetlands and stormwater ponds, especially those under heavy shade, provide an ideal environment for mosquito settlement. Ponds with plenty of sunshine and a shortage of vegetation are believed to be a poor environment for mosquito development [9]. WNV dissemination is found to be significantly related to average summer temperatures from 2002 to 2004 in the USA. [10].

Field and laboratory records of entomological and ecological observations have been used to examine how natural environmental constraints, such as water sources and climatic parameters, contribute to the transmission of WNV [11,12,9]. Doham and Turell (2001) found that the infection rates of WNV in mosquitoes are lower at cooler temperatures than when these vectors were maintained at warmer temperatures. The infection rates start to increase after one day of incubation at 26°C. WNV dissemination begins more rapidly in mosquitoes settled at higher temperatures than in mosquitoes maintained at cooler temperatures [12]. Gingrich et al. (2006) detected a bimodal seasonal distribution of mosquitoes with peaks in early and late summer in Delaware in 2004, and that mosquitoes are attracted to ponds with heavy shade and low slopes.

Remote sensing (RS) and geographic information system (GIS) technologies have been extensively applied in public health studies and related issues such as urban environmental analysis [13,6,5-20]. These technologies have been applied to research diverse epidemiological issues, such as parasitic diseases and schistosomiasis using RS and GIS as exclusive sources of information for studying epidemics. The accessibility of multi-temporal satellite imagery effectively supports the study of epidemiology [21]. Ruiz *et al*. (2004) found that some environmental and social factors contributed to WNV dissemination in Chicago in 2002 by using GIS technologies and multi-step Discriminant Analysis. Those factors included distance to a WNV positive dead bird specimen, the age of housing, the intensity of mosquito abatement, the presence of vegetation, geological factors, and demographic factors such as population age, income, and race. Multiple mapping techniques were compared for WNV dissemination in the continental USA [17]. The results indicated that each mapping technique emphasized certain WNV risk factor(s) due to the differences in modeling assumptions, statistic treatment, and error determination. There was no single model performing better than all others. Cooke III et al. (2006) estimated WNV risk in the state of Mississippi based on human and bird cases recorded in 2002 and 2003 with the creation of avian GIS models. The results indicate that high road density, low stream density, and gentle slopes contributed to the dissemination of WNV in Mississippi. GIS and spatial-time statistics were applied for a risk analysis of the 2002 equine WNV epidemic in northeastern Texas [19]. A total of nine non-random spatial-temporal equine case aggregations and five high-risk areas were detected in the study area. Ruiz et al. (2007) further examined the association of WNV infection and landscapes in Chicago and Detroit using GIS and statistical analysis. Their results show that higher WNV case rates occurred in the inner suburbs where housing ages were around 48–68 years old with moderate vegetation cover and population density.

Many valuable studies have documented the effects of environmental and socioeconomic factors on the spread of WNV. Despite this, a long-term study of these effects using data with high temporal resolution has yet to be undertaken. This study develops a multi-temporal analysis of the relationship between environmental variables and WNV dissemination using an integration of remote sensing, geographic information systems (GIS), and statistical techniques. The specific research objectives are to identify the spatial patterns of WNV outbreaks at different years and seasons in the city of Indianapolis, USA, to examine the relationships of WNV dissemination and environmental variables, and to investigate the temporal variations of the relationship. Through spatio-temporal analyses, it is possible to identify and explain the temporal outbreaks of WNV and the high-risk areas in the study area.

Indianapolis is a typical Midwest city lying in the flat plain and has a temperate climate without pronounced wet or dry seasons. Therefore, this study can offer not only valuable information for public health prevention and mosquito control, but also provide a testimony for the WNV spread in the other regions of the Midwest USA and beyond.

## Results

### Epidemic curves and outbreaks

**Figure 1:**
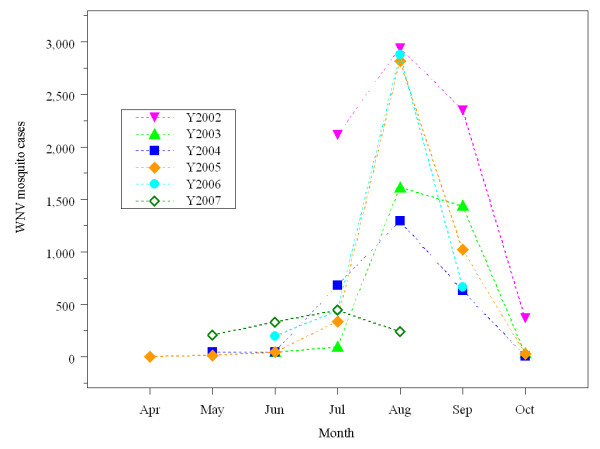
**Epidemic curves of mosquito WNV in 2002–2007**. X-axis shows individual months from April to October. Y-axis presents mosquito counts of WNV positive tests. Each curve represents WNV records in a single year from 2002 to 2007.

Figure 1 presents epidemic curves of WNV infections in mosquitoes from 2002 to 2007. In the figure, the Y-axis represents counts of WNV positive mosquitoes. Figure 1 shows a trend of increasing infections: in 2002 from July to October, in 2003 from June to October, in 2004 from May to October, and in 2005 from April to October. In 2006, records only cover the months of June through September and in 2007, the months of May through August. From 2002 through 2006, the epidemic curve reaches a peak in August. The exception is in 2007, where the peak is reached in July. The variations of curves are consistent with the ecological observation that WNV erupts in the summer and continues into the fall. We can conclude that the outbreak of WNV has been in August or July in the City of Indianapolis for the past six years. As for the spatial patterns of WNV outbreaks, WNV dissemination always starts from the central longitudinal corridor and spreads from there out to the east and west. This observation indicates that some environmental conditions may have significantly affected the spatial patterns of outbreaks. The results of Discriminant Analysis below provide important clues to explain this observation.

### Risk areas

**Figure 2:**
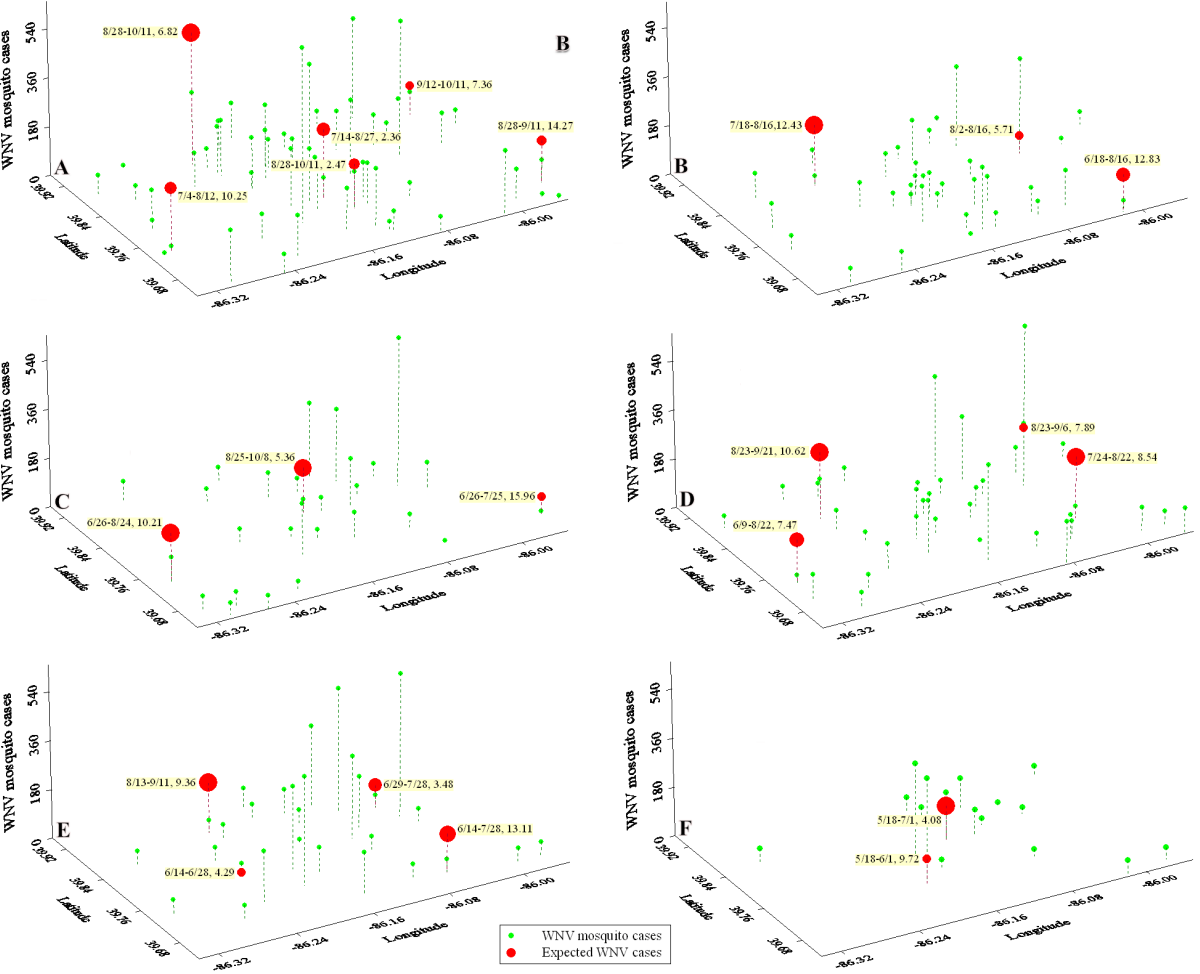
**WNV mosquito cases and their spatial-temporal clusters in years 2002–2007**. Figure 2A is for year 2002, and 2B for year 2003; Figure 2C is for year 2004, and 2D for year 2005; Figure 2E is for year 2006, and 2F for 2007. In each graph, X-axis presents latitude; Y-axis shows longitude; Z-axis shows WNV mosquito cases; Expected cases are shown in Z-axis for spatial-temporal clusters. Each cluster shows time period of cluster (month/day), cluster radius. p-value for rejecting the null hypothesis of no clustering is 0.001 for each cluster.

Spatial-temporal WNV clusters were detected in each year of 2002–2007 by using a Space-Time Permutation model in the SaTScan software application. According to Figure 2, year 2002 had six spatial-temporal clusters, 2003 and 2004 had three clusters, four clusters in 2005 and 2006, and two clusters in 2007. Each cluster had a specific time period of outbreak and individual radius. The p-value for rejecting the null hypothesis of no clustering is 0.001. Although these spatial-temporal clusters appeared randomly in space, multiple clusters can be found on the southeast corner of the study area in 2002–2006 with large radii (more than 8 km) and a time period ranging from June to September. Two clusters from two different years (2002 and 2004) even shared the same centroid (39.69 N, 85.95 W) but with different radii (14.27 km and 15.96 km) and the periods of outbreaks (8/28-9/11 and 6/26-7/25 respectively). The southeastern section of the city was dominated by agriculture and grassland. A high concentration of clusters with large radii indicates that agriculture and grassland provide favorable and extensive habitats for mosquito breeding during the summer and early fall. Three clusters in 2002, 2004, and 2005 were found to share the same centroid on the vegetation land in the central west of the city. This finding supports the conclusion that mosquitoes are attracted by grassland in the study area.

**Figure 3:**
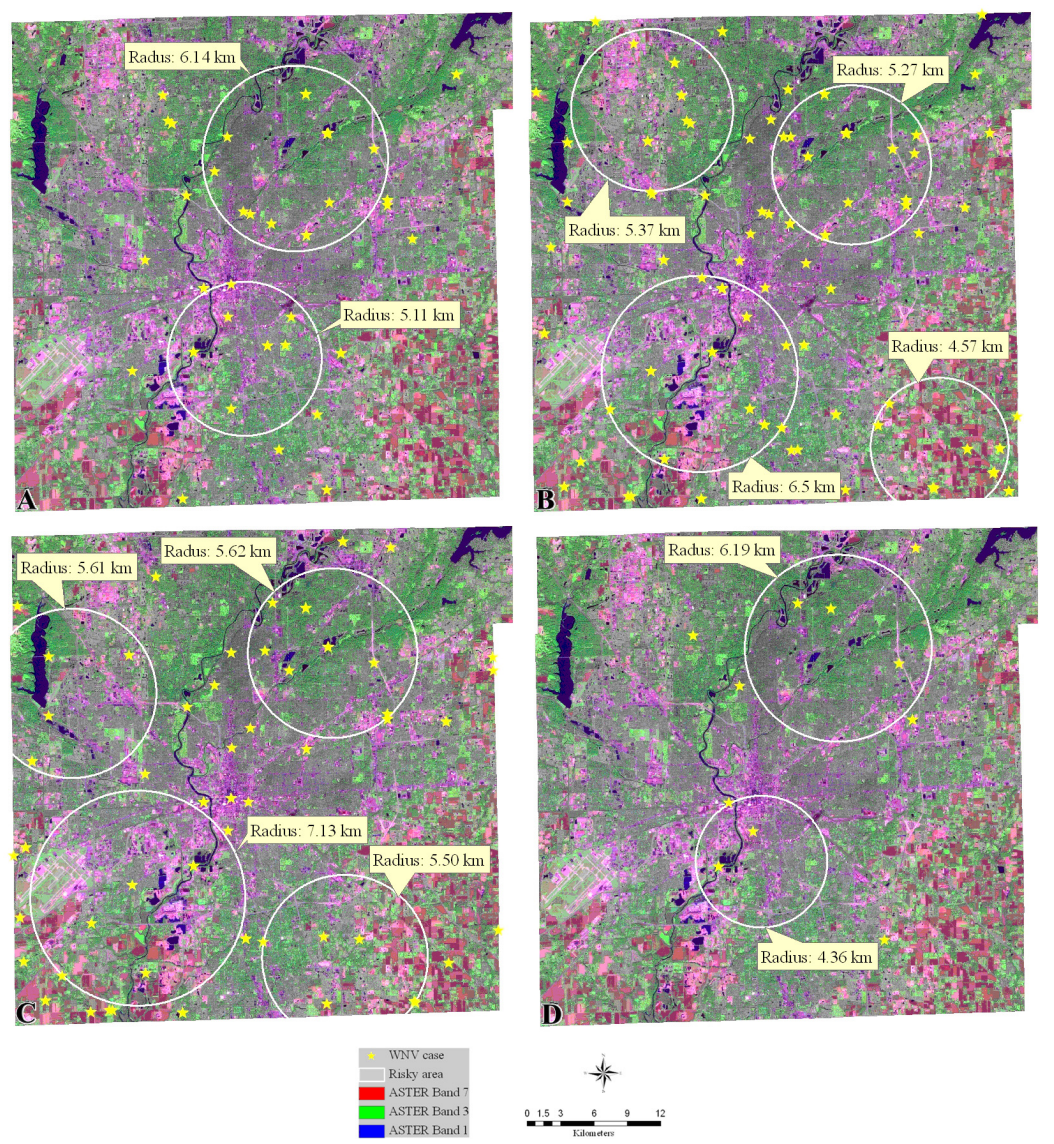
**Risky areas in August in years 2002–2007**. Figure 3A is for July; Figure 3B is for August; 3C is for September, and 3D is for October. The background image in each graph is an ASTER image with a false color composite (Band 7 in red, Band 3 in green, and Band 1 in blue).

The result of K-means cluster analysis on individual months of July, August, September, and October shows that different months possessed different high risk areas. There were two clusters in July, four clusters in August, four clusters in September, and two clusters in October in the study period. Figure 3 shows the high risk areas in the four months and their radii, which conforms to the explosive transmission of WNV in Indianapolis during the summer. In Figure 3, a cluster can be found in the southeastern corner of the city in each month of August and September. This finding supports the result of spatial-temporal analysis in which five WNV clusters were observed in the same area in 2002–2006. As noted in the previous paragraph, this area was dominated by crops (mainly corn and soybeans) and grass. In Indiana, corn is usually planted in April and May, and it becomes mature in September and October; while Soybeans are usually planted in May and June and is usually harvested in September and October. Dense corn and soybean fields in August and September provide suitable temperature and sufficient moisture for mosquito breeding, which might not happen in July when small leaves are still growing and neither in October when crops are harvested.

In Figure 3, two clusters appeared in the northwest part of the city in August and September. The August cluster was close to Eagle Creek reservoir, and the September one comprised the reservoir. According to records from a USGS streamflow gauging station, mean stream flow in the Eagle Creek Watershed was highest in March and lowest in September. Wetlands were expected to appear when the water level decreased from July to August and September, which provided favorable habitats for mosquito breeding combing with warm temperature in August and September. There was a cluster comprising dense ponds and part of the White River in the southeastern city in each month. A possible explanation to the observation is that the ponds and the river provided plenty of moisture and still water for mosquito breeding.

### Environmental Factors of WNV Dissemination

Table 1 shows environmental variables remained after the Discriminant Analysis, their coefficients, Eigenvalues, grouping accuracies, and the numbers of census block groups with mosquito WNV records and without mosquito cases. According to the result of the step-wise Wilks' lambda Discriminant Analysis, some environmental variables seem to be more effective than others to differentiate block groups with mosquito WNV cases from those without WNV records. The area percentage of agriculture was one of the most effective variables in Discriminant Analysis in all years except for year 2006. A higher proportion of agriculture land was associated with more WNV cases. This finding conforms to the risk area analysis in the section of Risk areas that agriculture lands in southeastern city were always within high-risk clusters in different years. It could be explained that agriculture helps to maintain constant moisture in the surrounding areas in the summer, which provided a favorable environment for mosquito breeding.

**Table 1:** Environmental factors remained after Discriminant Analysis.

**Year**	**Variable**	**Classification function coefficient**	**Eigenvalue**	**BG with WNV/BG without WNV**	**Grouping accuracy**
2002	Percentage of agriculture	0.143	0.226	46/612	86.90%
				
	Total length of streams	0.001			
				
	Total size of wetlands	0.001			
				
	Constant	-2.124			

2003	Percentage of agriculture	0.126	0.202	35/623	84.80%
				
	Total length of streams	0.001			
				
	Constant	-1.336			

2004	Percentage of agriculture	0.154	0.201	25/633	84.80%
				
	Percentage of water	0.037			
				
	Mean slope	1.908			
				
	DEM variation	0.049			
				
	Constant	-4.261			

2005	Percentage of agriculture	0.232	0.24	36/622	90.30%
				
	Total size of wetlands	0.001			
				
	Constant	-2.499			

2006	Total size of wetlands	0.001	0.157	29/629	85.30%
				
	Human population density	0.001			
				
	Constant	-2.141			

2007	Percentage of agriculture	0.051	0.195	16/642	88.90%
				
	Total length of streams	0.001			
				
	Percentage of water	0.205			
				
	Distance to the closest pollutant	0.001			
				
	Distance to the closest waste industry	0.003			
				
	Constant	-4.709			

Their coefficients, eigenvalue, the numbers of census blockgroups with mosquito WNV cases and without WNV cases, and grouping accuracies.

Total size of wetland was another important variable for WNV dissemination in the years of 2002, 2005 and 2006. Larger wetlands were linked to more mosquito WNV records. According to the regulations of US EPA, wetlands are those areas saturated by surface or ground water at a frequency and duration sufficient to support vegetation prevalence. A possible explanation to the observation is that high temperature and low to median precipitation in July and August in these three years created ideal conditions for the wetlands suitable for mosquito breeding.

Total length of streams plays an important role in mosquito WNV dissemination in years 2002, 2003, and 2007. Both stream density and the total length of streams were originally inputted to the Discriminant Analysis, but only the total length of streams remained after the analysis. Longer streams were associated with more WNV cases. This observation is in agreement with a WNV study in Mississippi which suggested that stream density contributed to WNV risk based on dead bird occurrences [18]. This result suggests that curvy streams with still water provided a favorable environment for mosquitoes breeding. Area percentage of water was remained after Discriminant Analysis for years of 2004 and 2007. Water information derived from ASTER imagery included big rivers, lakes, and dams. Higher percentage of water sources certainly contributed to more mosquito WNV cases.

In addition to agriculture, stream, wetland, and some other water sources, some other variables also showed a positive contribution to WNV dissemination in various years, including mean slope and elevation change for 2004; human population density in 2006; distance to the closest pollutant and distance to the closest waste industry for 2007. However, these variables were significant only for a specific year, not for the whole time period. According to the result of the Discriminant Analysis, high ground slope would be expected to contribute to the spread of WNV, which is contradictory to the fact that when surface slope is high, it is less possible to hold mosquito eggs and larvae during heavy rainfall. Human population density showed a positive contribution to WNV dissemination in 2006. It indicates that human behavior might have impacted the mosquito habitat in 2006. Distance to the closest pollutant and distance to the closest waste industry showed a positive contribution to the spread of WNV in 2007, which may indicate that there are well-built protection systems built in the pollutant and waste industry sites that eliminated mosquito breeding sites. Further studies are warrant in order to understand the complexity of environmental impacts on the WNV dissemination.

## Discussion

**Figure 4:**
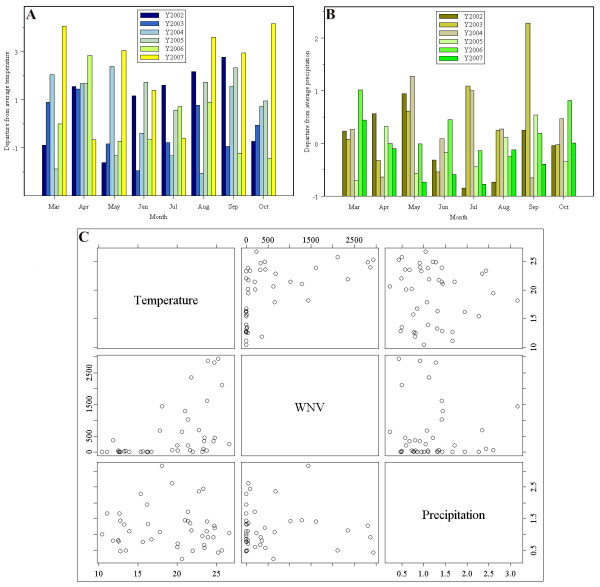
**Departure from average temperature and precipitation in Indianapolis in 2002–2007 from March to October**. Figure 4A shows the departure from average temperature in Indianapolis. Units: °C. Figure 4B presents the departure from average precipitation in Indianapolis. Units: meters. Figure 4C shows the relationships between temperature, precipitation, and WNV dissemination.

This study has conducted a spatio-temporal analysis of the relationship between WNV dissemination and environmental variables using the integration of remote sensing, GIS, and statistical techniques. Results indicate that although epidemic curves peak in the same month (i.e., August) from 2002 to 2006, the number of WNV cases varied by year and by month. In order to explain the temporal variations, two factors that were closely related to mosquito life cycling–particularly temperature and precipitation–were investigated based on the weather records of NOAA's National Weather Service Weather Forecast Office. The first two charts in Figure 4 present the monthly mean temperature and mean precipitation in 2002 and 2007 from March to October. The bottom chart in Figure 4 shows the relationship between temperature, precipitation, and the WNV cases. Low precipitation and warm temperature were found to associate with more WNV cases. Based on Figure 4, a possible explanation to the highest WNV counts in July in 2002 is that the year experienced the highest temperature and lowest precipitation in that month compared to any other year which provided an ideal natural environment for mosquito breeding. Years 2005 and 2006 had relatively higher temperatures and lower precipitations in August compared to those of years 2003 and 2004. This could help explain why both years had much higher WNV cases in August than those in 2003 and 2004. An obvious increase of rainfall from July to August in 2007 may contribute to lower WNV counts in August 2007 compared to those in July 2007. Other factors also need to be considered in order to completely understand the temporal variation of the WNV cases, especially any mosquito control and abatement measures done by local government or organizations.

## Conclusion

The research found that WNV dissemination always starts from central longitudinal corridor and spreads out to the eastern and western parts of the city. Different years and seasons had different high-risk areas. The southwestern and southeastern areas are believed to face the highest risk for WNV infection due to their high percentage of agriculture and water surfaces. Major environmental factors contributing to the outbreak of WNV in Indianapolis are high percentages of agriculture and water, the total lengths of streams, and the total size of wetlands. Other environmental factors, such as elevation variations, mean slope, human population density, and distances to the closest pollutant and waste industry need to be further examined in assessing their roles in the dissemination of WNV.

## Methods

### Study area

**Figure 5:**
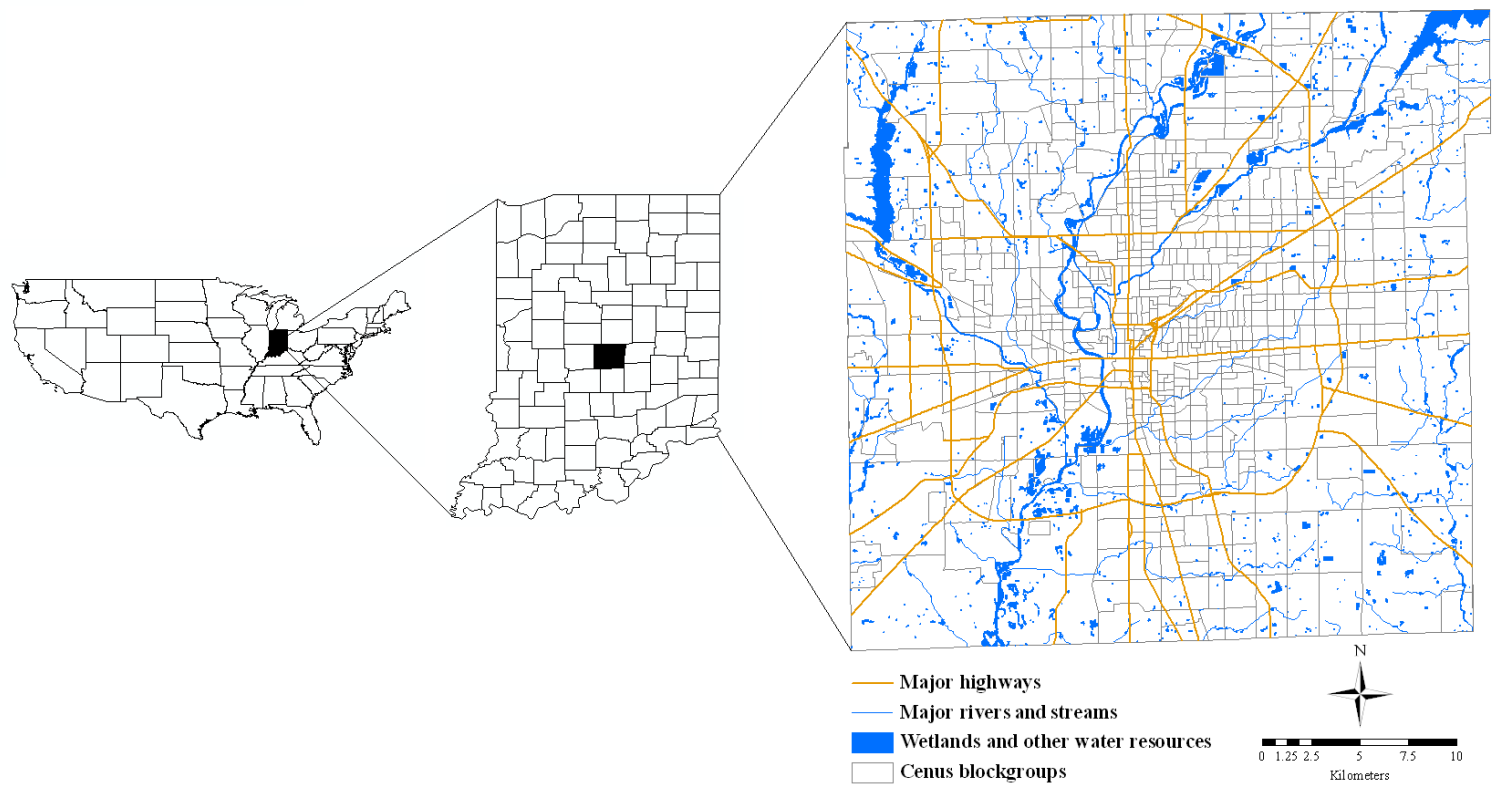
**Study area: Indianapolis, Indiana, USA**.

Indianapolis, USA, the capital city of Indiana and the seat of Marion County was chosen as the study area (Figure 5). It is the nation's twelfth largest city in population with about 800,000 in 2000. The Indianapolis metropolitan area had a population of 1.6 million in 2003. The whole city falls in a flat plain with an elevation change less than 60 meters. It has a temperate climate without pronounced wet or dry seasons. According the record of NOAA's National Weather Service Weather Forecast Office, its average high temperature is 1.39°C in January, 17.17°C in April, 29.78°C in July, and 18.67°C in October. The annual precipitation based on monthly average precipitation reaches 1.04 meters, in which May, June, and July have the highest records of monthly average precipitation with about 0.1 meters individually. January possesses the highest record of monthly average snowfall with 0.24 meters. Both December and February have the second highest record of 0.16 meters. The first WNV cases were reported only in birds in year 2001 in the city of Indianapolis. The first WNV infections in mosquitoes were recorded in July, 2002 and the first human cases were reported in the same year. According to the statistics of Centers for Disease Control and Prevention (CDC) , not as many human cases as mosquito cases have been documented since then in the study area: 41 human cases in 2002, two in 2003, one in 2004, two in 2005, five in 2006, and four human cases in 2007. This study demonstrates a temporal analysis by focusing on the WNV infections in mosquitoes in each month of all those years (2002–2007) that has WNV reports. Although the six-year period may be a short period of time for diseases that have been documented for decades or over a hundred of year (e.g., Acquired Immune Deficiency Syndrome and asthma), it can be considered as a long duration of time for WNV study because it first appeared in the study area since 2001 Figure 5.

### Data collection and processing

The WNV information was provided by the Indiana State Department of Health, which includes the physical locations of positive sites in the city of Indianapolis, USA, the number of positive mosquito pools in each of those sites, the number of mosquitoes in each pool, and the dates of sampling records. The time period is monthly from April through October in years 2002–2007. There was the only information available when the study was developed. Table 2 records the raw numbers of positive sites, positive mosquito pools in each positive site, and mosquitoes in each positive mosquito pool for years 2002–2007 in Indianapolis, USA. Since this study was to process a multi-temporal analysis, all positive sites in each year were geocoded and visualized based on their physical locations using ArcGIS, a popular program for GIS mapping and analysis. The geographical coordinates of positive sites were also calculated using the GIS software. Sites with positive WNV records in each month of July through October from 2002–2007 were combined and geocoded respectively to identify monthly variations. WNV records in April-June were not used for studying the monthly variations due to much fewer epidemic cases.

**Table 2:** The raw numbers of positive sites, positive mosquito pools in each positive site, and mosquitoes in each positive mosquito pool for years 2002–2007 in Indianapolis, USA.

**Year**	**WNV infection**		
	
	**Raw number of positive sites**	**Raw number of positive mosquito pools in each positive site**	**Raw number of mosquitoes in each positive mosquito pool**
2002	59	225	7807

2003	39	81	3228

2004	29	64	2722

2005	40	95	4266

2006	30	96	4181

2007	16	31	1230

**Figure 6:**
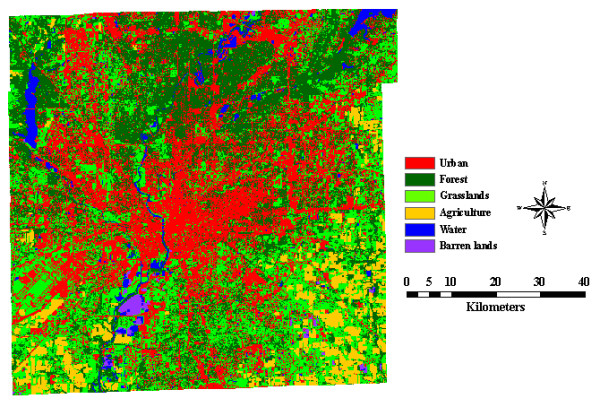
**Land use and land cover map of Indianapolis, Indiana, USA on October 13, 2006**.

Environmental factors, such as the presence of vegetation, the intensity of mosquito abatement measures, the age of housing, and steam density were believed to have contributed to the dissemination of WNV [6,18,19]. Some of those factors were used in this study and additional factors were added to the analysis due to data accessibility and local environmental conditions. Land use and land cover (LULC) information was derived from three Advanced Spaceborne Thermal Emission and Reflection Radiometer (ASTER) images acquired on June 16, 2001, April 5, 2004, and October 13, 2006, respectively. The spatial resolution for the visible bands is 15 m, 30 m for the near infrared bands, and 90 m for the thermal infared bands. Unsupervised image classification method was chosen to classify the image into six categories: urban, agriculture, forest, grasslands, water, and barren lands. Overall accuracy of image classification reached 88.33%, 92.0%, and 89.0%, individually. Figure 6 shows the October image as an example. It becomes known from the image that urban, forest, and grassland were dominant habitats, with urban areas dominant in the central part of the city and the forest areas mainly located in the north mixed with grassland. Agriculture was concentrated in the southeastern and southwestern parts of the city. There were two major reservoirs, the Geist Reservoir and the Eagle Creek Reservoir located in the northern parts of the study area. The White River runs north-south through the city.

Other environmental data were collected from the Indiana Geological Survey website . Data collected from there include the estimated percentages of impervious surfaces in Indiana in 2001; a digital elevation model with 1.5-meter resolution; an inventory of hydrogeologic terrains and settings; national wetland inventory data; pipe locations in the National Pollutant Discharge Elimination System (NPDES); facilities in NPDES; and an inventory of industrial waste sites in Indiana. The outlines of the census block groups were also downloaded from the same website. There were a total of 658 census block groups in this study area. This study was based on an assumption that selected environmental variables in a certain census block group contributed the most to the WNV dissemination in that blockgroup. As a result, all factors were summarized and analyzed at the census blockgroup level. A centroid of each census blockgroup was calculated by using ArcGIS. The shortest distances from each centroid to the pollutants and industrial waste sites were calculated. The total length of streams and the area of wetlands in each census blockgroup were also computed in ArcGIS. The stream density was calculated as a ratio between the total length of streams in a census blockgroup and the area of the same census blockgroup. Human population density in each census blockgroup was selected to examine the possible influence of human behavior to the spread of WNV. Table 3 lists all the variables used in the study.

**Table 3:** The environmental variables selected for this study. Study unit: census block groups.

**Variable**	**Description**	**Mean for all 658 census blockgroups in Indianapolis, Indiana, USA**.
Land use land cover (LULC)	Area percentage of each LULC category	Varied by seasons and years

Impervious surface	Average percentage of impervious surface	38.8%

Elevation	Elevation variation	12.9 m (42.3 ft)

Slope	Mean slope	1.6°

Stream	Total stream length and stream density	466.7 m (1531.2 ft)

Wetland	Total size of wetlands	51437.4 m^2 ^(553 667.6 sq.ft)

Pollutant	Distance from centroid of each census blockgroup to the closest pollutant in National Pollutant Discharge Elimination System (NPDES)	3,201.8 m (10 504.6 ft)

Pipe	Distance from centroid of each census blockgroup to the closest pipe in NPDES	7,831.3 m (25,693.2 ft)

Waste industry	Distance from centroid of each census blockgroup to the closest waste industry site	804.9 m (2,640.7 ft)

Population density	Human population density	1,587 per sq. km

### Plotting epidemic curves

WNV was first identified in Indiana in 2001 and its transmission was enhanced during the summer of 2002. Culex pipiens was the main vector mosquito in the state. Six cumulative epidemic curves were created to show the peaks and the temporal trends of mosquito WNV outbreaks in Indianapolis in years 2002 to 2007. Epidemic patterns could be identified based on monthly and annual comparison. The spatial outbreaks of WNV were tracked to indicate the movement of WNV dissemination in the last six years.

### Risk area estimation

It is significant to identify the risk areas of WNV mosquito outbreaks so that special care could be taken. A retrospective Space-Time Permutation model in SaTScan software was selected to identify non-random WNV clusters in years of 2002–2007. This spatial-temporal model was based on a null hypothesis that there were no spatial, temporal, or spatial-temporal clusters in the study area. The p-value for the most likely cluster would be larger than 0.05 with a 95% change. The Monte Carlo hypothesis testing was used to calculate the test statistic for all possible clusters and 999 random replications. If a possible cluster was among the 5% highest, then the significant level of test is 0.05 [22]. The pre-defined maximum spatial cluster size was 50 percent of the population at risk, and the pre-defined maximum temporal cluster size was 50 percent of the study period. The spatial window shape was elliptical with medium non-compactness penalty. The centroids and radii of the most likely clusters with p-value less than 0.01 were recorded in a DBF table and then visualized by using ArcGIS.

A K-means cluster analysis was developed to identify the high-risk areas for each month of July, August, September, and October. In order to increase the sampling size in the statistical analysis, the records collected in the same month but different years were combined. Three variables were selected from each location: the coordinates of the location, the number of positive mosquito pools in each month, and total number of mosquitoes in those pools. The maximum iterations were set to be ten with zero convergence criterions. The centers and radii of high-risk areas in different months (July, August, September, and October) were identified based on the results of the cluster analysis. The Analysis of Variance (ANOVA) table helped to examine which variables are the most important in the cluster analysis and the number of cases in each cluster was used to make sure the proportional sizes of the clusters.

### Discriminant analysis

Discriminant analysis is very useful for determining which variables discriminate between two or more groups and it helps to build a predictive model of group membership based on observed characteristics of each case [23]. Its analysis result is a multiple regression equation(s) and those variables that contribute most to the discrimination of group membership are the ones with the largest standardized coefficients. Discriminant analysis is less flexible than regression analysis because it requests the independent variables to be normally distributed and not equal variance within each group [24]. Some environmental variables are believed to have more influence than others in the spread of WNV [6,7,11,12]. By using the Discriminant analysis, those environmental variables could be identified in the study area. A step-wise Wilks' lambda Discriminant analysis was applied to compare the census blockgroups with WNV cases to those without any cases relative to the set of environmental factors in Table 1. There were 658 census blockgroups used in this study. The analysis was done by year.

## Competing interests

The authors declare that they have no competing interests.

## Authors' contributions

HL conducted the research and wrote the first draft of the manuscript. QW provided expertise in research design and revised the manuscript. DG provided expertise on WNV and mosquitoes.
